# Mesenchymal Stem Cells and NF-κB Sensing Interleukin-4 Over-Expressing Mesenchymal Stem Cells Are Equally Effective in Mitigating Particle-Associated Chronic Inflammatory Bone Loss in Mice

**DOI:** 10.3389/fcell.2021.757830

**Published:** 2021-10-14

**Authors:** Ning Zhang, Takeshi Utsunomiya, Tzuhua Lin, Yusuke Kohno, Masaya Ueno, Masahiro Maruyama, Ejun Huang, Claire Rhee, Zhenyu Yao, Stuart B. Goodman

**Affiliations:** ^1^Department of Orthopaedic Surgery, Stanford University, Stanford, CA, United States; ^2^Department of Bioengineering, Stanford University, Stanford, CA, United States

**Keywords:** wear particles, chronic inflammation, bone loss, mesenchymal stem cells, macrophages

## Abstract

Wear particles from total joint arthroplasties (TJAs) induce chronic inflammation, macrophage infiltration and lead to bone loss by promoting bone destruction and inhibiting bone formation. Inhibition of particle-associated chronic inflammation and the associated bone loss is critical to the success and survivorship of TJAs. The purpose of this study is to test the hypothesis that polyethylene particle induced chronic inflammatory bone loss could be suppressed by local injection of NF-κB sensing Interleukin-4 (IL-4) over-expressing MSCs using the murine continuous polyethylene particle infusion model. The animal model was generated with continuous infusion of polyethylene particles into the intramedullary space of the femur for 6 weeks. Cells were locally injected into the intramedullary space 3 weeks after the primary surgery. Femurs were collected 6 weeks after the primary surgery. Micro-computational tomography (μCT), histochemical and immunohistochemical analyses were performed. Particle-infusion resulted in a prolonged pro-inflammatory M1 macrophage dominated phenotype and a decrease of the anti-inflammatory M2 macrophage phenotype, an increase in TRAP positive osteoclasts, and lower alkaline phosphatase staining area and bone mineral density, indicating chronic particle-associated inflammatory bone loss. Local injection of MSCs or NF-κB sensing IL-4 over-expressing MSCs reversed the particle-associated chronic inflammatory bone loss and facilitated bone healing. These results demonstrated that local inflammatory bone loss can be effectively modulated via MSC-based treatments, which could be an efficacious therapeutic strategy for periprosthetic osteolysis.

## Introduction

Wear particles from total joint arthroplasties (TJAs) incite a persistent macrophage-mediated chronic inflammatory reaction resulting in the release of cytokines, chemokines, and other molecules (Bi et al., 2001; Xu et al., 2009; Abu-Amer, 2013) and, stimulate key paracrine and autocrine cell interactions (Goodman et al., 2013). This reaction promotes bone resorption and impedes bone formation, leading to periprosthetic osteolysis and eventually, loss of mechanical support for the implant (Goodman, 2007; Goodman et al., 2014; Qiu et al., 2020). Revision surgery for osteolysis is technically demanding, with higher costs and poorer outcomes than primary arthroplasty (Kurtz et al., 2014). Although modern bearing couples and alloys have been developed to reduce wear, new strategies for reducing particle-induced osteolysis are needed to improve the durability of TJAs.

Mesenchymal stem cells (MSCs) have shown great potential in skeletal tissue regeneration (Caplan, 1991; Zhang et al., 2021). Previously we showed an intervention using unaltered MSCs during the chronic inflammatory phase could mitigate the adverse effects of contaminated particles on bone (Utsunomiya et al., 2021a). Furthermore, specific properties of MSCs, such as differentiation capability and immunomodulation potential can be further refined by genetic modification to optimize MSC-based therapy (Wei et al., 2018; Zhang et al., 2021). Whether local delivery of genetically modified MSCs could abrogate the adverse effects of particles on bone *in vivo*, using the murine continuous femoral infusion model is unknown. Interleukin-4 over expression by genetically modified MSCs mitigates inflammation by converting pro-inflammatory M1 macrophages to an anti-inflammatory M2 phenotype (Lin et al., 2017b); modulation of macrophage phenotype at an appropriate time can optimize osteogenic differentiation of MSCs (Lin et al., 2019) and enhance bone regeneration (Chow et al., 2019; Niu et al., 2021). An *in vitro* study showed that genetically modified MSCs over-secreting IL-4 trigged by NF-κB activation could mitigate the proinflammatory response of macrophages exposed to wear particle (Lin et al., 2018). In the present study, we injected NF-κB sensing IL-4 over-expressing MSCs locally in the murine continuous femoral particle infusion model. We test whether NF-κB sensing IL-4 over-expressing MSC treatment is a more efficacious therapeutic strategy than unaltered MSCs for particle induced chronic inflammatory bone loss in this model.

## Materials and Methods

### Cells

Male BALB/c murine bone marrow derived MSCs were isolated and characterized as previously described (Lin et al., 2015). Briefly, 8–10- week-old BALB/c male mice were used to collect the bone marrow from femurs and tibias. The bone marrow with cells was filtered through 70 μm cell strainer, spun down and resuspended using α-minimal essential medium (α-MEM, Thermo Fisher Scientific, Waltham, MA United States) supplied with 10% certified fetal bovine serum (FBS, Invitrogen, Thermo Fisher Scientific, Waltham, MA United States) and antibiotic-antimycotic solution (100 units of penicillin, 100 μg of streptomycin and 0.25 μg of amphotericin B per milliliter, Hyclone, Thermo Fisher Scientific, Waltham, MA United States). Unattached cells were removed by replacing medium the next day (passage 1). Flow cytometry (LSRII, Stanford Shared FACS Facility, Stanford, CA, United States) was used to characterize the immunophenotype of isolated MSCs at passage 4: spinocerebellar ataxia type 1 (Sca1^+^)/CD105^+^ /CD44^+^ /CD34^–^/CD45^–^/CD11b^–^. Identified MSCs passages 4–8 were used in the experiments. This protocol has been approved by Stanford’s Administrative Panel on Laboratory Animal Care (APLAC).

### Generation of Genetically Modified Mesenchymal Stem Cells

The lentiviral vector preparation was performed as previously described (Pajarinen et al., 2015). Human embryonic kidney 293T cells (ATCC, Manassas, VA, United States) were used to transfect the control lentivirus vector the mouse IL-4 secreting pCDH-NF-κB-mIL-4-copGFP expressing lentivirus vector (Lin et al., 2017b) together with psPAX2 packaging vector and pMD2G VSV-G envelope vector using the calcium phosphate transfection kit (Clontech, Mountain View, CA, United States) with 25 μM chloroquine. The virus was diluted in MSC culture medium supplemented with 6 μg/ml of polybrene (Sigma Aldrich, St. Louis, MO, United States), and infected to murine MSCs at multiplicity of infection (MOI) = 100. At 3 days post-infection, the infected cells were GPF positive confirmed by fluorescence microscope (Keyence, Itasca, IL, United States).

### Enzyme-Linked Immunosorbent Assay

ELISA kits for mouse IL-4 (R&D system, Minneapolis, MN, United States) were used to quantify IL-4 expression by the genetically modified MSCs exposed to 1 μg/ml LPS (Sigma Aldrich, St. Louis, MO) or left untreated for 24 h culture. The manufacturers’ protocols were carefully followed. The optical densities were determined using SpectraMax M2e Microplate Readers (Molecular Devices, San Jose, CA, United States) set at 450 nm with wavelength correction set to 540 nm.

### Ultra-High Molecular Weight Polyethylene Particles

The polyethylene particles were processed as previously described (Lin et al., 2018, 2019). Briefly, Ceridust 3,610 polyethylene particles (Clariant Corporation, CA, United States) were washed by ethanol and filtered using a 20 μm pore membrane. A particle size of 4.62 ± 3.76 μm was verified by an electron microscopy (Cell Science Image Facility at Stanford University). The filtered particles were vacuum dried for 3 days then resuspended using Phosphate-Buffered Saline (PBS) containing 5% Bovine Serum Albumin (BSA, Thermo Fisher Scientific). The concentration of the resuspended particles was approximately 3.1 × 10^10^ particles/ml. 10 ng/ml of lipopolysaccharides (LPS, Sigma-Aldrich St. Louis, MO, United States) was used to generate the contaminated particles (cPE) (Greenfield et al., 2010). The endpoint chromogenic Limulus Amebocyte Lysate assay (Lonza, Portsmouth, NH, United States) was used to confirm the sterility of the particles.

### Continuous Femoral Infusion Murine Model

**FIGURE 1:**
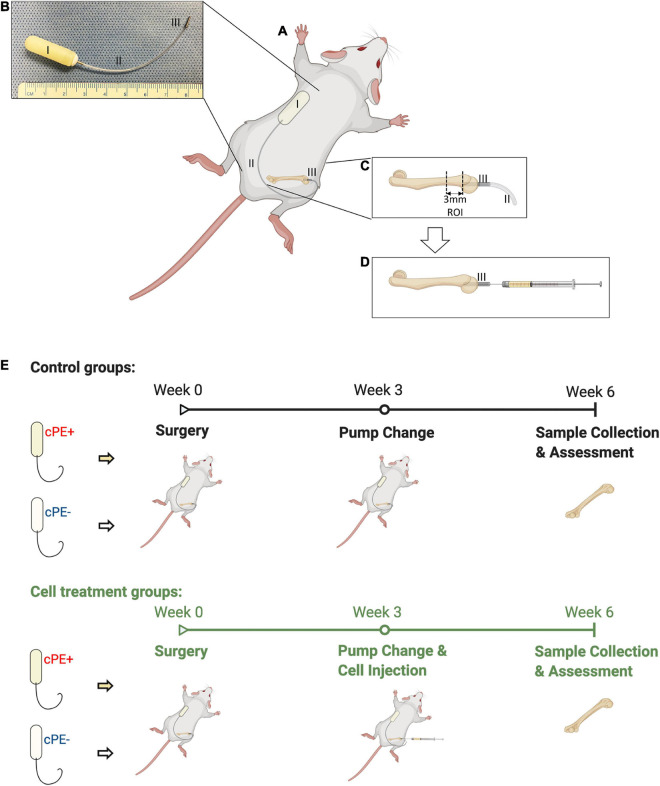
Continuous femoral infusion murine model generation and experimental design based on the treatments. **(A)** The murine continuous femoral infusion model. **(B)** Infusion system. I: osmotic pump; II: connected tube; III: hollow titanium rod. **(C)** Rod press-fit into the distal femoral canal and the selected ROI for assessments. **(D)** Cell injection at 2nd surgery. **(E)** Summary of the treatments and experimental design.

The animal experiment was approved by the Institutional APLAC at Stanford University (Protocol number: 17566). Institutional Guidelines for the Care and Use of Laboratory Animals were followed in all aspects of this project. Eleven to twelve-week-old BALB/c male mice were used to generate the murine continuous femoral infusion model as previously described (Ma et al., 2008; Ren et al., 2011; Gibon et al., 2012; Sato et al., 2015, 2016; Lin et al., 2016; Nabeshima et al., 2017; Pajarinen et al., 2017; Goodman et al., 2019). The surgery was conducted on mice under preoperative analgesia by subcutaneously injection of 0.1 mg/kg of buprenorphine, and inhalation anesthesia using 1 L/min flow of 2% isoflurane in 100% oxygen on small animal surgery station at 37°C. The right distal femur was exposed after patellar dislocation via a lateral parapatellar incision. 25 gauges, 23 gauges, and 22 gauges needles were used sequentially to pierce through the intercondylar notch into the medullary cavity. A hollow titanium rod (6 mm long, 23 gauge) (Figures 1A–D, part III) was then press-fit into the distal canal of the femur. An osmotic pump (Figures 1A–D, part I) containing 10% BSA/PBS with or without contaminated polyethylene particles (cPE, 1.25% of polyethylene particles and 10 ng/ml of LPS) was implanted into dorsal side of mouse subcutaneously through a second incision around the right shoulder girdle; the pump was connected to the implanted rod via subcutaneous vinyl catheter tubing (Figures 1A–D, part II). Skin incisions were closed using 5–0 Ethilon sutures (J&J Medical Devices) after all the procedures. No infection was observed in any of the mice and all mice appeared to ambulate relatively normally several days after the surgical procedure. Three weeks after primary surgery, pumps (Figures 1A–D, part I) and connected tubing (Figures 1A–D, part II) were removed. 10 μl PBS with 0.5 × 10^6^ MSCs or NF-κB sensing IL-4 over-expressing MSCs were injected through the rod into the femur (Figures 1A–D, part III), then pumps and tubes were changed to new ones containing 10% BSA/PBS with/without cPE, which was infused for 3 more weeks (Figure 1E).

Thus, there were 6 groups with/without cPE and with/without different MSCs as follows: (1) control without cPE (cPE- control group), (2) MSCs injection at 3 weeks after primary surgery without cPE (cPE- MSCs group), (3) NF-κB sensing IL-4 over-expressing MSCs injection at 3 weeks after primary surgery without cPE (cPE- IL-4 MSCs group), (4) control with cPE (cPE + control group), (5) MSCs injection at 3 weeks after primary surgery with cPE (cPE + MSCs group), (6) NF-κB sensing IL-4 over-expressing MSCs injection at 3 weeks after primary surgery with cPE (cPE + IL-4 MSCs group).

### Micro-Computational Tomography

**FIGURE 2:**
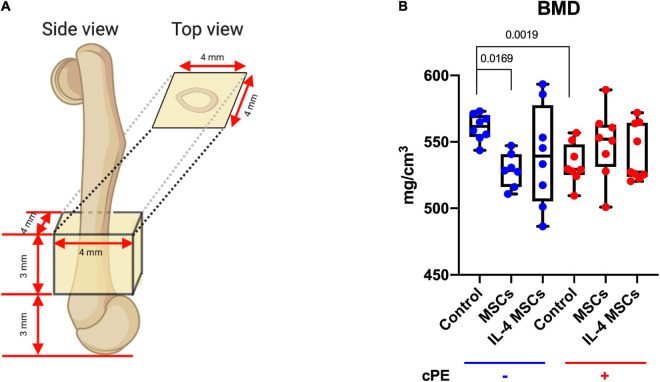
Bone Mineral Density (BMD) within the ROI for all groups. **(A)** The 3D ROI was defined as a 4 mm × 4 mm × 3 mm box and started 3 mm from the distal femur end. **(B)** Quantitative assessments of BMD within the region of interest for all groups. (cPE- control group: *n* = 8; cPE- MSCs group: *n* = 7; cPE- IL-4 MSCs group: *n* = 8; cPE + control group: *n* = 8; cPE + MSCs group: *n* = 8; cPE + IL-4 MSCs group: *n* = 9).

Mice were euthanized 6 weeks after the primary surgery (Figure 1E) and the titanium rod was removed from the distal femur. μCT scans were performed using TriFoil Imaging CT120 (TriFoil Imaging, Chatsworth, CA, United States) with 49 μm resolution. A 4 mm × 4 mm × 3 mm three-dimension (3D) region of interest (ROI) was created within the distal femur which began 3 mm from the distal end of the femur and proceeded proximally (Figures 1C, 2A; Lin et al., 2016; Nabeshima et al., 2017). The threshold bone mineral density (BMD, mg/mm^3^) was quantified by using GEMS MicroView software (threshold: 700 HU).

### Tissue Processing and Histological Staining

Isolated femurs were fixed with 4% paraformaldehyde overnight and decalcified with 0.5 M ethylenediamine tetra acetic acid (EDTA, pH 7.4). After dehydration, the specimens were embedded in optimal cutting temperature (OCT) compounds. The ROI (Figure 1C) located 3 mm from the distal end of femur was cut into transverse sections with 10 μm-thickness for subsequent staining (Lin et al., 2016). Hematoxylin and Eosin (H&E) staining and immunohistochemistry were performed for general tissue morphological assessment and morphometry.

### Immunohistochemistry for Macrophage Phenotype Analysis

To identify the macrophages by immunohistochemistry, the sections were covered by blocking buffer (5% BSA) for 30 min at room temperature followed by primary and secondary antibody incubation for 1 h each at room temperature. Macrophages were identified by immunofluorescence staining with rat anti-mouse F4/80 monoclonal antibody (CI: A3-1, Bio-Rad) followed by Alexa Fluor^®^ 594 conjugated goat anti-rat IgG (Invitrogen, CA, United States). M1 pro-inflammatory macrophages were further identified by rabbit anti-mouse inducible nitric oxide synthase (iNOS) polyclonal antibody (Abcam, Cambridge, MA, United States) followed by Alexa Fluor^®^ 488 conjugated goat anti-rabbit IgG (Invitrogen, CA, United States). M2 anti-inflammatory macrophages were identified by rabbit anti-mouse liver Arginase (Arg1) polyclonal antibody (Abcam, Cambridge, MA, United States) followed by Alexa Fluor^®^ 488 conjugated goat anti-rabbit IgG (Invitrogen, CA, United States). ProLong Gold Antifade Mount with DAPI (Life Technologies, Grand Island, NY, United States) was used to mount the slides. A fluorescence microscope (Digital Microscope, Keyence, IL, United States) was used to detect the immunohistochemistry staining. Finally, the cells were manually counted in 3 randomly selected fields of view by Image J software (National Institutes of Health, United States).

### Osteoclast-Like Cells and Osteoblast-Like Cells Detection

Osteoblast-like and osteoclast-like cells were identified as previously described (Goodman et al., 2013; Sato et al., 2015; Miron et al., 2016; Brooks et al., 2019). The tartrate resistant acid phosphatase (TRAP) staining kit (Sigma-Aldrich, St. Louis, MO, United States) was used to identify osteoclast-like cells; multi-nucleated TRAP positive cells located on the bone perimeter within the resorption lacunae were defined as osteoclast-like cells. For the detection of osteoblast-like cells, anti-alkaline phosphatase staining (1-stepTM NBT/BCIP Substrate Solution, Thermo Fisher Scientific, Rockford, IL) was used. The TRAP positive cell number of 6 randomly selected areas in each section and the percentage of ALP positive area of the entire area of each section were quantified using Image J software (National Institutes of Health, United States) according to our previous protocol (Utsunomiya et al., 2021a). The color threshold of each parameter was determined by consensus of three of the investigators. Double-blinded quantitative analysis was conducted by two of the investigators.

### Statistical Analysis

The statistical analysis was conducted using Prism 8 (GraphPad Software, San Diego, CA, United States). Data were expressed as median with interquartile range. Mann-Whitney *U*-test was performed to evaluate the difference between the control group with and without cPE. The Kruskal-Wallis test with Dunn’s multiple comparisons was used to compare data with 3 or more groups. *p* < 0.05 was regarded as statistically significant.

## Results

### NF-κB Sensing Interleukin-4 Over-Expressing Mesenchymal Stem Cells Generation and Characterization

**FIGURE 3:**
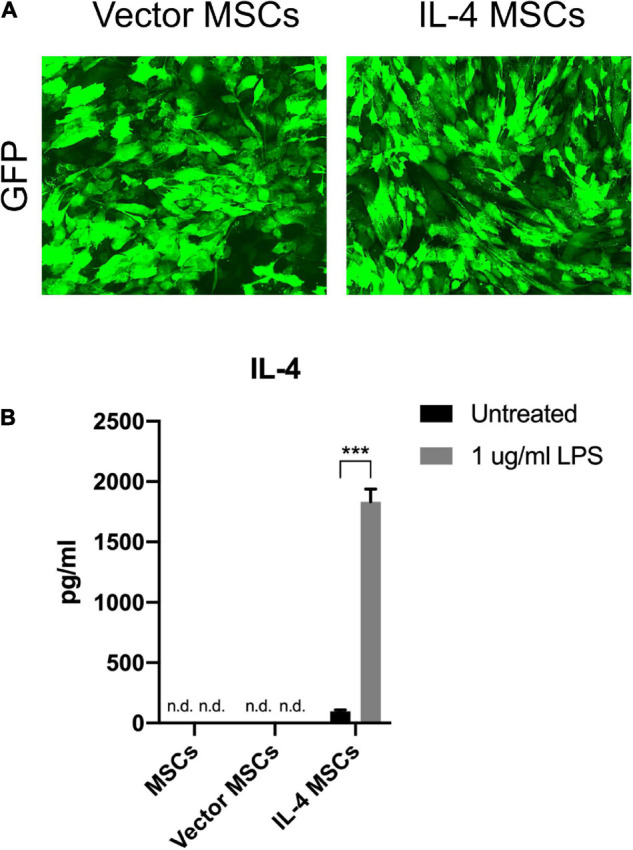
Characterization of MSCs and genetically modified MSCs. **(A)** Representative fluorescence microscopy images of genetically modified MSCs. **(B)** IL-4 secreting levels detected by ELISA in the culture media with or without 1 μg/ml LPS treatment for 24 h (n.d.: cannot be detected by ELISA; ***: *p* < 0.001). Note the high levels of IL-4 protein produced by the IL-4 MSCs exposed to LPS.

Murine MSCs were successfully infected by empty lentiviral vectors (Vector MSCs) and NF-κB sensing IL-4 expressing lentiviral vectors (IL-4 MSCs). GFP positive MSCs were also produced successfully (Figure 3A). The IL-4 secretion in the unaltered MSC group and Vector MSC group was below the detectable range by ELISA with or without 1 μg/ml LPS treatment (Lin et al., 2017b). The current LPS concentration (1 μg/ml) was used according to the protocols of previous studies investigating the effect of LPS on MSCs with the goal of reliably inducing NF-κB activation rather than modeling any specific disease state (Pevsner-Fischer et al., 2007; Wang et al., 2009; Ti et al., 2015). IL-4 secretion in the IL-4 MSCs group was significantly upregulated by 1 μg/ml LPS treatment for 24 h (from 97.75 ± 11.29 pg/ml to 1832.55 ± 105.19 pg/ml, Figure 3B).

### Local Injection of Cells Decreased M1 Macrophage and Increased M2 Macrophage Proportions in the Presence of Contaminated Particles

**FIGURE 4:**
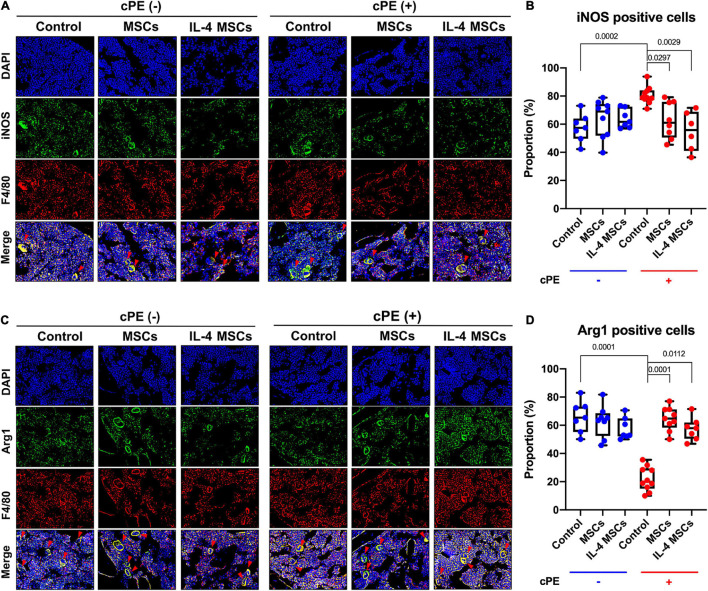
Immunohistochemistry and quantitative analysis for M1 and M2 macrophages in all groups. **(A)** Representative images of immunohistochemistry for M1 macrophages (iNOS + , F4/80 + , red arrows). **(B)** Quantitative analysis for iNOS positive cell proportion (cPE- control group: *n* = 7; cPE- MSCs group: *n* = 9; cPE- IL-4 MSCs group: *n* = 7; cPE + control group: *n* = 10; cPE + MSCs group: *n* = 8; cPE + IL-4 MSCs group: *n* = 6). **(C)** Representative images of immunohistochemistry for M2 macrophages (Arg1 + , F4/80 + red arrows). **(D)** Quantitative analysis for Arg1 positive cell proportion (cPE- control group: *n* = 7; cPE- MSCs group: *n* = 8; cPE- IL-4 MSCs group: *n* = 7; cPE + control group: *n* = 10; cPE + MSCs group: *n* = 9; cPE + IL-4 MSCs group: *n* = 7).

The proportion of M1 pro-inflammatory macrophages (iNOS+, F4/80+) in cPE + control group was significantly increased compared with that in cPE- control group (*p* = 0.0002) (Figures 4A,B). Local injection of MSCs significantly decreased the proportion of M1 macrophages when comparing cPE + MSCs group with cPE + control group (*p* = 0.0297). Local injection of NF-κB sensing IL-4 over-expressing MSCs also significantly decreased the proportion of M1 macrophages when comparing cPE + IL-4 MSCs group with cPE + control group (*p* = 0.0029) (Figures 4A,B). The M2 anti-inflammatory macrophage (Arg1+, F4/80+) proportion in cPE + control group was significantly decreased compared with that in cPE- control group (*P* = 0.0001) (Figures 4C,D). Local injection of MSCs and NF-κB sensing IL-4 over-expressing MSCs significantly increased the proportion of M2 macrophages when comparing cPE + MSCs group with cPE + control group (*p* = 0.0001) and comparing cPE + IL-4 MSCs group with cPE + control group (*p* = 0.0112) (Figures 4C,D).

### Local Injection of Cells Reduced the Osteoclast-Like Cell Number Induced by Contaminated Particles

**FIGURE 5:**
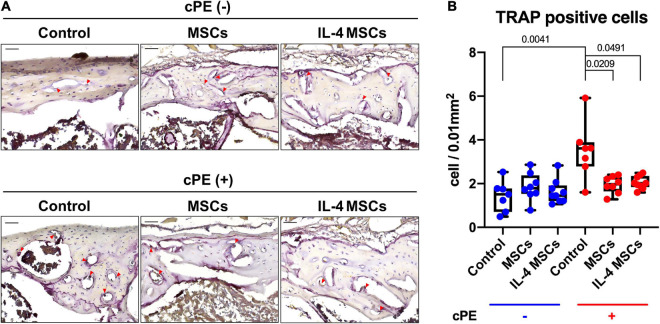
TRAP staining of all the groups. **(A)** Representative images of TRAP staining, red arrows point the TRAP positive cells. **(B)** Quantitative analysis of TRAP positive cells number. (cPE- control group: *n* = 7; cPE- MSCs group: *n* = 8; cPE- IL-4 MSCs group: *n* = 9; cPE + control group: *n* = 7; cPE + MSCs group: *n* = 8; cPE + IL-4 MSCs group: *n* = 9).

The TRAP staining positive cell number in cPE + control group was significantly increased compared with that in the cPE- control group (*p* = 0.0041) (Figures 5A,B). Local injection of cells including MSCs and NF-κB sensing IL-4 over-expressing MSCs significantly reduced the TRAP positive cell number in cPE + MSCs group and cPE + IL-4 MSCs group when compared with the cPE + control group (*p* = 0.00209, *p* = 0.0491, respectively). No differences were observed among cPE- groups with or without injection of cells (Figures 5A,B).

### Local Injection of Cells Increased the Positive Staining Area of Osteoblast-Like Cells

**FIGURE 6:**
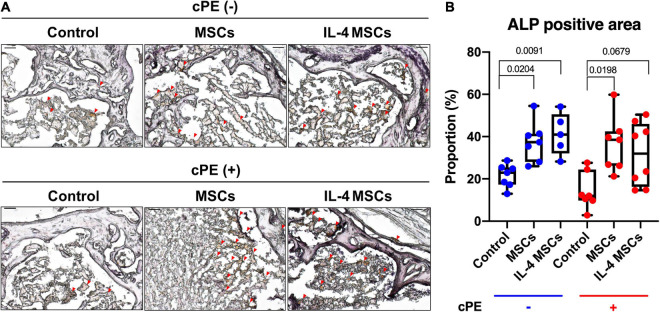
ALP staining of all the groups. **(A)** Representative images of ALP staining, red arrows point the ALP positive area. **(B)** Quantitative analysis of ALP positive proportion. (cPE- control group: *n* = 7; cPE- MSCs group: *n* = 7; cPE- IL-4 MSCs group: *n* = 5; cPE + control group: *n* = 7; cPE + MSCs group: *n* = 7; cPE + IL-4 MSCs group: *n* = 8).

The percentage of ALP positive area in cPE + control group showed decreased staining compared with that in the cPE- control group, but the decrease did not reach statistical significance (Figures 6A,B). The percentage of ALP positive area in cPE + MSCs group was significantly (*p* = 0.0198) increased compared with that in the cPE + control groups (Figure 6B) and the percentage of ALP positive area in the cPE + IL-4 MSCs group exhibited a strong trend (*p* = 0.0679) when compared with that in the cPE + control group (Figure 6B). The percentage of ALP positive area in the cPE- MSCs group increased significantly compared with that in the cPE- control group (*p* = 0.0204). The percentage of ALP positive area in the cPE- IL-4 MSCs group demonstrated a dramatic increase (*p* = 0.00091) compared with that in the cPE- control group (Figure 6B).

### Local Injection of Mesenchymal Stem Cells Mitigates Contaminated Particles-Induced Bone Loss in Chronic Inflammation

The BMD in the ROI (Figure 2A) of the cPE + control group was significantly reduced compared with the BMD of the cPE- control group (*p* = 0.0019). Local injection of MSCs and NF-κB sensing IL-4 over-expressing MSCs in the presence of cPE increased the BMD compared with the cPE + control group although no significant differences were detected. Interestingly, after local injection of MSCs in the cPE- groups, the cPE- MSCs group showed significantly lower BMD compared with the cPE- control group (*p* = 0.0169). The cPE- IL-4 MSCs group also showed slightly lower BMD compared with the cPE- control group, but no significant difference was detected (Figure 2B).

## Discussion

Chronic inflammation around implants due to byproducts of wear or other causes (e.g., instability, low grade infection etc.) is still a major an unsolved problem. We have demonstrated that local delivery of contaminated polyethylene particles (cPE) can induce acute or chronic inflammation and polarize macrophages to a pro-inflammatory M1 rather than an anti-inflammatory M2 macrophage phenotype in different *in vitro* and *in vivo* models (Utsunomiya et al., 2021a, b). Macrophages are the characteristic cell type involved in chronic inflammation (Allison et al., 1978; Maruyama et al., 2020); furthermore modulation of macrophage phenotype at an appropriate time can optimize osteogenic differentiation of MSCs (Lin et al., 2019) and the enhancement of the bone regeneration (Chow et al., 2019; Niu et al., 2021). The increased pro-inflammatory M1 macrophage proportion and the decreased anti-inflammatory M2 macrophage proportion caused by cPE over a 3-week period was subsequently reduced by the local injection of MSCs and NF-κB sensing IL-4 over-expressing MSCs at harvest 3 weeks later. The increased TRAP positive osteoclast-like cell number, the lower ALP positive osteoblast-like area and BMD trend in the presence of cPE infusion further confirmed that the cPE could induce bone loss (Lin et al., 2016, 2017a; Pajarinen et al., 2017; Utsunomiya et al., 2021a, b). The injection of MSCs and NF-κB sensing IL-4 over-expressing MSCs reversed these findings in part.

The results of TRAP staining (Figure 5) showed significant differences between control groups with and without cPE. However, only a trend was noted when assessing ALP staining comparing cPE- control group with cPE + control group. Similar results concerning the TRAP and ALP staining were observed in other reports using the murine continuous infusion model with polyethylene particles (Lin et al., 2016, 2017a). This suggests that the cPE might selectively affect the regulation of osteoclastogenesis more than osteoblastogenesis. Therapeutic strategies targeting the regulation of osteoclastogenesis could be a promising approach to limit the bone loss due to wear particles. Interestingly, similar efficacy was observed between controls vs. the MSCs or IL-4 MSCs group in the presence of cPE, respectively. It would appear that unaltered MSCs are sufficiently activated to downregulate the chronic inflammation induced by cPE.

The presence of cPE significantly reduced BMD in the control groups. Previous reports showed that injection of MSCs and preconditioning of MSCs during the chronic inflammation stage could increase the BMD in the presence of cPE (Utsunomiya et al., 2021a). In the current study, local injection of MSCs and NF-κB sensing IL-4 over-expressing MSCs also tended to increase BMD in the presence of cPE. Surprisingly, local injection of MSCs reduced the BMD without cPE. The possible reasons for these observations may be that this is a shorter-term model of a complex process, and BMD may be less sensitive than other methods of analysis to determine efficacy using the parameters chosen for this experiment. In fact, we observed confirmatory differences in the histological and immunohistological results, which are consistent with our previous *in vitro* studies. Possible reasons for the lack of difference in BMD may be the specific shorter-term model used, the particle load chosen, the well-established and efficacious immunomodulatory properties of MSCs alone and the production of IL-4 by the MSCs. A longer time period for particle infusion and resultant bone loss, and for subsequent cell delivery may further highlight the potential efficiency of MSCs and NF-κB sensing IL-4 over-expressing MSCs in the murine cPE infusion model.

Nuclear factor kappa-light-chain- enhancer of activated B cells (NF-κB) is the key transcription factor associated with chronic inflammation and osteolysis (Xu et al., 2009; Abu-Amer, 2013; Zuo et al., 2018). Previous studies have shown that suppression of NF-κB by its decoy oligodeoxynucleotides (ODNs) could enhance osteogenesis in MSCs exposed to polyethylene particles *in vitro* (Lin et al., 2015) and mitigate the *in vivo* particle induced chronic inflammatory osteolysis (Lin et al., 2016, 2017a; Utsunomiya et al., 2021b). In the current study, NF-κB sensing IL-4 secreting MSCs produced IL-4 when the NF-κB pathway was activated by inflammatory signals; IL-4 production ceased when the inflammatory activation signal was withdrawn (Lin et al., 2017b). IL-4 was only expressed by the genetically modified MSCs during the ongoing chronic inflammation period, limiting potential adverse effects caused by excessive IL-4 expression (Lin et al., 2017b; Zhang et al., 2021). This feedback mechanism would be potentially useful in other inflammatory conditions in other organ systems.

Surprisingly, the unaltered MSCs were as effective as the NF-κB sensing IL-4 over-expressing MSCs in reversing the adverse effects of cPE on bone. This may be due to the specific animal model chosen, and the duration and particle load delivered. The efficacious immunomodulatory properties of MSCs by themselves with/without the over-production of IL-4 may be the potential reason for this comparable effectiveness. More prolonged and intense inflammatory stimuli may demonstrate an augmented utility of NF-κB sensing IL-4 over-expressing MSCs in chronic inflammation.

The current study has some limitations. Unaltered MSCs were locally injected rather than MSCs infected with an empty vector (vector-MSCs) to investigate the efficacy of naïve MSCs. Six weeks of continuous cPE infusion is a relatively shorter-term time period compared to the longer-term process associated with periprosthetic osteolysis in clinical scenarios. Only one time point at week 6 analysis was conducted; multiple time points for analysis would be more informative about this process. The generation of MSCs with combinations of co-expressing cytokines, growth factors, or chemokines to enhance the bone regeneration (Nabeshima et al., 2017; Zhang et al., 2020, 2021) in the presence of wear particles might be more efficient in mitigating the particle induced chronic inflammatory bone loss.

In summary, continuous infusion of cPE into the femur increased the pro-inflammatory M1 macrophage phenotype and decreased the anti-inflammatory M2 macrophage phenotype. Also, cPE increased osteoclastogenesis and lowered BMD. Local delivery of either MSCs or NF-κB sensing IL-4 over-expressing MSCs is a potential therapeutic intervention in mitigating particle-associated chronic inflammatory bone loss.

## Data Availability Statement

The raw data supporting the conclusions of this article will be made available by the authors, without undue reservation.

## Ethics Statement

The animal study was reviewed and approved by the Stanford’s Administrative Panel on Laboratory Animal Care (APLAC).

## Author Contributions

NZ and TU contributed to the study design, acquisition, analysis and interpretation of data, drafting and revision of the manuscript. TL, YK, MU, MM, EH, and CR contributed to the acquisition and analysis and interpretation of data. ZY contributed to the study design and interpretation of data. SG contributed to the study conceptualization and design, interpretation of data, and critical revision of the manuscript. All authors approved the submission of the manuscript.

## Conflict of Interest

The authors declare that the research was conducted in the absence of any commercial or financial relationships that could be construed as a potential conflict of interest.

## Publisher’s Note

All claims expressed in this article are solely those of the authors and do not necessarily represent those of their affiliated organizations, or those of the publisher, the editors and the reviewers. Any product that may be evaluated in this article, or claim that may be made by its manufacturer, is not guaranteed or endorsed by the publisher.
